# Daily Perceived Stress and Physical Health Symptoms: Moderation by Self-Perceptions of Aging

**DOI:** 10.1093/geroni/igab046.2311

**Published:** 2021-12-17

**Authors:** Dakota Witzel, Shelbie Turner, Karen Hooker

**Affiliations:** 1 Oregon State University, Corvallis, Oregon, United States; 2 Oregon State University, Oregon State University, Oregon, United States

## Abstract

Research suggests increased daily perceived stress is related to worse physical health outcomes such as poor eating and exercise behavior (Li et al., 2019) and lower perceived health (Whitehead & Blaxton, 2020). While long-term implications for increased perceptions of stress on physical health are becoming clear (e.g., Rueggeberg et al., 2012), less is known about associations between daily perceptions of stress and subjective levels of physical health symptoms. Moreover, positive perceptions of one’s own aging may buffer perceived stress’s impact on physical health. Indeed, self-perceptions of aging (SPA) impact how people prepare for age-related stressors (Kornadt et al., 2015), and are associated with physical health trajectories (Luo & Li, 2020). Using a 100-day microlongitudinal study of 103 older adults, we examined the 1.) impact of both between- and within-persons associations of perceived stress on physical health symptoms and 2.) the potential moderating associations of SPA on perceived stress and physical health symptom associations. Preliminary models suggest that on days when people perceived more stress, they show a .03 increase in reporting physical health symptoms compared to days when they do not report more perceived stress (p<.0001). Further, people who experience more perceived stress on average, reported .06 more physical symptoms across the study period (p <.0001). While SPA did significantly predict physical health symptoms (p=.004), the association between perceived stress and physical health symptoms was not dependent on SPA (p>.05). Future directions may include exploring associations between daily stressful experiences, perceptions of stress, and valence of SPA.

